# Evaluating tumor chemosensitivity: a head-to-head comparison of the prognostic value of KELIM (modeled CA125 elimination rate constant K) and RECIST 1.1 (radiological response valuation criteria in solid tumors) in ovarian cancer

**DOI:** 10.1007/s00404-025-08285-z

**Published:** 2026-02-19

**Authors:** Kaja Michalczyk, Agata Mokrzycka, Marianna Rudzińska, Marcin Misiek, Anita Chudecka-Głaz

**Affiliations:** 1 Department of Gynecological Surgery and Gynecological Oncology of Adults and Adolescents, Pomeranian Medical University, Szczecin, Poland; 2 Collegium Medicum, University of Zielona Gora, Zielona Gora, Poland; 3 Department of Gynecologic Oncology, Holy Cross Cancer Center, 25-734 Kielce, Poland

**Keywords:** Ovarian cancer, Chemotherapy, KELIM, CA-125, RECIST

## Abstract

**Purpose:**

The aim of the study was to analyze KELIM (modeled CA125 ELIMination rate constant K) and RECIST 1.1. (radiological response valuation criteria in solid tumors) as indicators of tumor chemosensitivity and their role in predicting patient prognosis.

**Methods:**

This retrospective single-center analysis included 165 consecutive patients with advanced newly diagnosed high-grade serous ovarian, fallopian tube, or primary peritoneal cancer who underwent surgical and chemotherapeutical treatment at the Department of Gynecologic Oncology.

**Results:**

There were significant differences in OS between the neoadjuvant and adjuvant groups of patients (20.87 vs 32.88 months). There was a significant difference in the response to treatment assessed in imaging studies between the groups, with higher rates of complete and partial responses to treatment among PDS patients (*p* = 0.002). However, upon a separate analysis of the NACT and PDS subgroups, the multivariate analysis showed no significant influence of KELIM and RECIST 1.1. response on patients’ overall survival of patients.

**Conclusion:**

Our findings showed no significant associations between KELIM, RECIST and overall survival of patients. However, further studies on bigger homogenous population samples are required to confirm our findings.

## What does this study add to the clinical work

**Table:** 

Although patients undergoing primary surgery had better survival outcomes than those receiving neoadjuvant chemotherapy, neither the KELIM score nor RECIST 1.1 radiological response proved to be significant predictors of overall survival in this study. Further research with larger populations is needed to determine the true prognostic value of these chemosensitivity indicators.	

## Introduction

The standard of treatment for newly diagnosed advanced ovarian cancer consists of cytoreductive surgery and platinum-based chemotherapy. For years, it has been discussed in which configuration (surgery or chemotherapy first) the treatment should be performed and if it affects patient outcomes. However, little information and efforts were made to determine the impact of chemotherapy efficacy among such patients. During the last few years, multiple indicators of tumor chemosensitivity were reported by different authors, concentrating mainly on pathological response score, tumor biomarkers, presence of tumor genomic alternations, DNA scar imprinting, and treatment response in medical imaging. Further studies revealed the importance of tumor primary chemosensitivity as a prognostic factor, i.e., the feasibility of performing complete tumor cytoreduction after neoadjuvant chemotherapy, its association with the risk of subsequent platinum-resistant relapse, progression-free survival, overall survival, or the efficacy of use of maintenance therapies including anti-angiogenic or PARP-inhibitor treatment.

As most of the patients present with advanced stages of the disease at the time of diagnosis (up to 65–80% of patients diagnosed as FIGO III and IV [1]), the decision between primary surgery and NACT is often challenging. Due to the strong prognostic value of complete cytoreduction during the debulking surgery, the disease management guidelines advise to perform the cytoreductive surgery at time when the likelihood of completed cytoreduction, with no residual disease, is sufficiently high and can be either performed as a primary debulking surgery, or as an interval debulking procedure [2, 3].

KELIM and radiological response valuation criteria in solid tumors (RECIST 1.1) are used to evaluate ovarian cancer treatment. However, they aim to assess different aspects of treatment response. KELIM (modeled CA125 ELIMination rate constant K) is a mathematical longitudinal kinetics model accounting for ≥ 3 CA125 biomarker values assessed during the first 100 days of chemotherapy treatment, either adjuvant or neoadjuvant [4]. While RECIST 1.1 evaluation focuses on visible tumor changes during cancer treatment, providing insight into direct tumor shrinkage, KELIM allows us to get some information about the effectiveness of treatment at a cellular level.

The study aimed to analyze KELIM and RECIST as indicators of tumor chemosensitivity and their role in predicting patient prognosis.

## Methods

This retrospective study included 165 consecutive patients with advanced newly diagnosed high-grade serous ovarian, fallopian tube, or primary peritoneal cancer, FIGO stage III and IV. All of the included patients underwent surgical and chemotherapeutical treatment at the Department of Oncological Gynecology between June 2018 and April 2023. The last follow-up was performed by the end of February 2024. Patients who did not undergo cytoreductive surgical treatment at the department or patients with incomplete clinicopathological data were excluded from the study. Patients’ age, tumor histology, CA125 concentration, BRCA mutation status (Next Generation Sequencing, Illumina Miseq), type of chemotherapy (neoadjuvant/ adjuvant), surgery type (PDS/ IDS) and its outcomes (optimal/ suboptimal cytoreduction/ no surgery), and computed tomography response assessed in accordance to RECIST 1.1 criteria were collected. KELIM (CA-125 elimination rate constant K) was calculated based on the longitudinal kinetics of CA-125, with at least three CA-125 measurements during the first 100 days of chemotherapy treatment (at the time of 1st, 2nd, and 3rd admission for chemotherapy either in adjuvant or neoadjuvant setting), either in the neoadjuvant or adjuvant setting. Its values were dichotomized with standard KELIM cutoff, dividing patients into unfavorable if std KELIM is less than 1.0 or favorable if the value is equal or greater than 1.0. Due to the observational nature of the study, the Pomeranian Medical University Research Ethics Committee has confirmed that no ethical approval is required, KB.006.139.2025.

In accordance with the journal’s guidelines, we will provide our data for independent analysis by a selected team by the Editorial Team for the purposes of additional data analysis or for the reproducibility of this study in other centers if such is requested.

## Results

The final analysis included 123 patients who met the study inclusion criteria. Among the analyzed sample, the mean age of patients at diagnosis was approximately 63 years, with a relatively wide range of values, from approximately 25 to almost 84 years. Out of the population sample, 51 patients (41.5%) underwent neoadjuvant chemotherapy, while 72 (58.5%) received adjuvant treatment. Most patients were classified as stage III according to the FIGO classification (101 patients), while 22 patients were diagnosed as FIGO IV. Furthermore, most of the study population did not carry any *BRCA* mutations. 15 patients had a *BRCA*1 mutation in the NGS test, while 10 patients were found to have a *BRCA*2 mutation.

Regarding response to treatment according to imaging, the dominant group was patients with partial response. When partial response and complete response were combined into one category and stable disease and progressive disease into another, a nonrandom distribution was also obtained. The KELIM index showed a similar share of people with unfavorable and favorable values, without significant differences in randomness. Detailed results of the distributions of the study subgroups, together with statistics for the univariate χ2 test, are presented in Table 1.

**Table 1:** Patients’ characteristics

		Number of patients	%	χ^2^	*p*
Type of chemotherapy treatment	Neoadjuvant	51	41.5%	3.58	0.058
	Adjuvant	72	58.5%		
BRCA type 1	No	98	86.7%	60.97	< 0.001
	Yes	15	13.3%		
BRCA type 2	No	103	91.2%	76.54	< 0.001
	Yes	10	8.8%		
Residual disease	R = 0	40	33.3%	0.80	0.670
	R < 1 cm	44	36.7%		
	R ≥ 1 cm	36	30.0%		
KELIM	< 1	61	53.5%	0.56	0.454
	≥ 1	53	46.5%		
Computed tomography response	Partial response	61	52.1%	57.60	< 0.001
	Stable disease	20	17.1%		
	Complete response	31	26.5%		
	Progressive disease	5	4.3%		
Computed tomography response	Partial response + complete response	92	78.6%	38.37	< 0.001
	Progressive disease + stable disease	25	21.4%		

^*^ Residual disease R0- no macroscopic disease, optimal cytoreduction: residual tumor < 1 cm; suboptimal cytoreduction: residual tumor ≥ 1 cm; x^2^ – chi squared statistics; p- probability

The distribution of qualitative demographic variables was compared between the neoadjuvant and adjuvant treatment groups. Variables, such as BRCA mutation status, residual disease after cytoreductive surgery, and the dichotomized KELIM index, did not show significant differences between the treatment groups. There was a significant difference in the response to treatment assessed in imaging studies. In the adjuvant group, complete response was several times more frequent; it occurred in over 87% of complete response cases, while in the neoadjuvant treatment group, it was noted in only about 13% of patients with this type of response. On the contrary, disease stabilization or progression occurred much more often in the neoadjuvant group. After combining progressive disease and stable disease responses into one category and complete response and partial response into another, significant differences were still visible; in the neoadjuvant group, about 68% of patients achieved progressive disease and stable disease response, while in the adjuvant treatment group, complete response and partial response responses predominated, accounting for 66% of this group. The complete results are demonstrated in Table 2.

**Table 2:** Comparison of qualitative demographic variables between the neoadjuvant and adjuvant treatment groups

			NEOADJUVANT		ADJUVANT		
		N	%	N	%	*χ*^2^	*p*
BRCA total	No	40	43.5%	52	56,5%	1.57	0.210
	Yes	6	28.6%	15	71,4%		
BRCA type 1	No	43	43.9%	55	56.1%	3.07	0.080
	Yes	3	20.0%	12	80.0%		
BRCA type 2	No	41	39.8%	62	60.2%	0.39	0.531
	Yes	5	50.0%	5	50.0%		
Residual disease	R = 0	16	40.0%	24	60.0%	0.54	0.764
	R < 1 cm	16	36.4%	28	63.6%		
	R ≥ 1 cm	16	44.4%	20	55.6%		
KELIM	Unfavorable	23	37.7%	38	62.3%	0.04	0.834
	Favorable	21	39.6%	32	60.4%		
Computed tomography response	Partial response	27	44.3%	34	55.7%	20.56	0.000
	Stable disease	12	60.0%	8	40.0%		
	Complete response	4	12.9%	27	87.1%		
	Progressive disease	5	100.0%	0	0.0%		
Computed tomography response	Partial response + complete response	31	33.7%	61	66.3%	9.49	0.002
	Progressive disease + stable disease	17	68.0%	8	32.0%		

^*^ *N- Number of patients;* x^2^ – chi squared statistics; p- probability

Multivariate logistic regression analysis were performed to determine the influence of specific variables on overall survival (Table 3). The analyses were performed separately for the neoadjuvant and adjuvant subgroups of patients. For the adjuvant population, none of the variables are statistically significant at the *p* < 0.05 level. Age above median, KELIM > 1, and R above 0 have hazard ratios less than 1, suggesting a potential decreased risk, but these are not statistically significant. FIGO 4 and mut BRCA 1 and 2 have hazard ratios greater than 1, suggesting a potential increased risk, but these are not statistically significant. For the neoadjduvant population, also none of the variables are statistically significant. The hazard ratios for all variables are close to 1, and the confidence intervals are relatively wide, indicating a lack of strong association between these predictors and survival time in this dataset.

**Table 3:** Cox regression analysis for OS

	Variable	Hazard Ratio (HR)	95% Confidence Interval (CI)	*P*-value
ADJUVANT	Age (above vs below median)	0.779	(0.279–2.172)	0.634
	FIGO (IV vs III)	2.381	(0.244–23.228)	0.474
	BRCA mutation vs no mutation	2.298	(0.729–7.251)	0.159
	R = 0 vs R ≥ 1	0.397	(0.129–1.223)	0.107
	KELIM < 1 vs ≥ 1	0.679	(0.228–2.023)	0.488
	TK response PD/ SD vs PR/CR	0.999	(0.000–1.359)	0.999
NEOADJUVANT	Age (above vs below median)	0.999	(0.995–1.004)	0.747
	FIGO (IV vs III)	1.158	(0.688–1.947)	0.576
	BRCA mutation vs no mutation	0.984	(0.517–1.871)	0.960
	R = 0 vs R ≥ 1	0.881	(0.511–1.519)	0.652
	KELIM < 1 vs ≥ 1	0.802	(0.418–1.538)	0.513
	TK response PD/ SD vs PR/CR	1.194	(0.619–2.302)	0.588

### Survival analysis

**Fig. 1:**
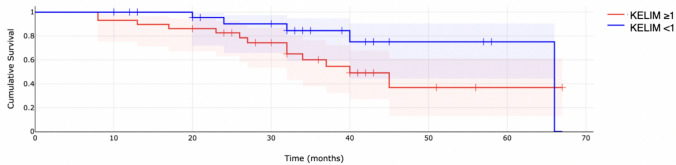
Overall survival for adjuvant chemotherapy patients

**Fig. 2:**
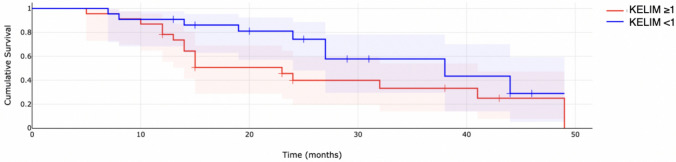
Overall survival for neoadjuvant chemotherapy patients

Survival analysis was performed using Kaplan–Meier analysis and the log-rank test. The estimated median overall survival for the whole study population was approximately 39.60 months. For the adjuvant chemotherapy population, the median OS was 32.88 months. When analyzed based on the KELIM score (KELIM < 1 vs ≥ 1), median OS for patients with KELIM < 1 was 32.33 months, and 33.90 months for KELIM ≥ 1; however, the differences were not statistically significant (p = 0.091). Similarly, for patients undergoing neoadjuvant chemotherapy followed by interval debulking surgery, median OS for was 20.87 months. For patients with KELIM < 1, the median OS was 15.57 months, and 25.00 months for KELIM ≥ 1; however, also in this population, the differences were not significant (*p* = 0.140). Kaplan–Meier survival curves were then presented according to the KELIM constant (classified as favorable vs. unfavorable) separately for the neoadjuvant and adjuvant populations of patients (Figs. 1, 2).

## Discussion

Multiple indicators of tumor primary chemosensitivity were proposed to help determine the primary response to systematic treatment and ease the therapeutic decision process with objective, measurable markers. The most common include the pathology examination (chemotherapy response score from surgical specimen [2, 6], level of tumor-infiltrating lymphocytes [7, 8]), use of genomic biomarkers (*BRCA*1 or 2 mutation testing[9, 10], homologous recombination status testing [11], multi-gene signature assays [12, 13]), imaging studies using radiological response [14, 15] and circulating markers assessment (KELIM kinetics [4, 16], circulating tumor cells [17], circulating tumor DNA [18], CA125, HE4 (human epididymis protein 4) [19, 20]).

In this study, we evaluated the use of KELIM kinetics and radiological treatment response assessed by RECIST 1.1. with regard to patients’ prognosis. Survival analysis showed significant differences in overall survival between the neoadjuvant and adjuvant populations of patients (20.87 vs 32.88 months). Thus, separate analysis was performed to assess the association between OS and KELIM value. Multivariable analysis showed no significant differences in overall survival, when stratified for KELIM neither in the adjuvant nor in neoadjuvant setting. In the adjuvant chemotherapy population, median OS for patients with KELIM < 1 was 32.33 months, and 33.90 months for KELIM ≥ 1(*p* = 0.091). Similarly, for patients undergoing neoadjuvant chemotherapy followed by interval debulking surgery, for patients with KELIM < 1, the median OS was 15.57 months, and 25.00 months for KELIM ≥ 1(*p* = 0.140). Surprisingly, despite the significant differences in CT response between the patients in PDS and IDS subgroups, with patients in the adjuvant group having significantly better responses to first-line chemotherapy in medical imaging, upon multivariate analysis, the CT response (complete/ partial response vs stable/progress disease) did not significantly influence patients’ overall survival. However, due to the retrospective nature of the study, and limited number of patients evaluated in the study, the results should be interpreted with caution as the results might have been affected by patient selection bias associated with the single institute patient cohort. So far, there are limited data regarding the possible application or radiological response criteria in predicting patients' outcomes. Bogani et al. conducted a study to evaluate the application of RECIST 1.1. response in ovarian cancer patients undergoing neoadjuvant chemotherapy. The authors showed radiological response to neoadjuvant treatment to impact the inability to perform complete cytoreduction at the time of IDS. Moreover, patients showing complete or partial response in RECIST criteria had better disease-free survival. However, the radiological response did not predict patients' overall survival [15]. While RECIST 1.1. is widely used as a standard for assessing tumor response, it has several limitations. Despite being objective, with strict assessment tumor assessment guidelines, it primarily focused on unidimensional measurements, which can underestimate the actual tumor shrinkage or growth, especially in tumors of an irregular shape, thus not accurately reflecting treatment-induced changes in tumor volume and heterogeneity. This may potentially cause discrepancies between RECIST 1.1 assessment and patients’ clinical outcomes[21, 22]. One of the problems is also the poor sensitivity of the techniques to asses peritoneal micro-infiltrative ovarian cancer lesions[23]. RECIST cannot also be used in patients with no measurable disease as it primarily focuses on target lesions selected during the baseline study. Non-target lesions, which are not included in the baseline study assessment, can be challenging to manage and may not be accurately represented in the RECIST assessment [24, 25].

The developed KELIM kinetics model allows for the characterization of CA125 dynamics in both treatment settings (PDS and IDS), and its reliability allows for the use of KELIM as an independent indicator of tumor chemosensitivity. The reproducibility has been shown in multiple clinical trials including CALYPSO [16], AGO-OVAR 7 [26], AGO-OVAR 9 [26], ICON-7 [27], and CHIVA [4] trials. However, as they were clinical trials, the populations of patients were preselected and thus more homogenous, creating less bias when compared to real-world studies. Research confirms the role of KELIM in the prediction of the likelihood of complete resection at the time of interval cytoreductive surgery [4, 28, 29], determination of the probability of subsequent platinum-resistant relapse [4, 30], and showing longer progression-free and overall survival in patients with favorable KELIM when compared to patients with unfavorable KELIM [4, 16, 26]. However, so far, most of the studies were based on retrospective analysis of clinical trials. In our study, in the survival analysis, KELIM, when used as a dichotomized value, either favorable or unfavorable, did not reach statistical significance as a variable influencing patients' progression-free or overall survival. Also, the KELIM score had a limited predictive value in multivariate analysis, which may be due to relatively small comparison subgroups. In the whole study, the progression-free survival was significantly shorter than overall survival and most often fell below 50% in the first two years of follow-up limiting the number of patients with more extended observation periods. KELIM score and response in imaging studies had a weaker but noticeable association with the risk of recurrence, especially in cases of achieving complete response.

There are very limited studies that concomitantly evaluated multiple indicators of tumor chemosensitivity. A retrospective analysis of CHIVA trial showed a gradual association between increasing std KELIM values and higher radiological tumor response in patients undergoing neoadjuvant chemotherapy [4]. A study by Piedimonte et al. demonstrated a correlation between the KELIM score and the chemo-response score (CRS) as chemosensitivity predictors. Patients with higher surgical pathologic CRS score (CRS3) and favorable KELIM score (≥ 1) had significantly higher PROGRESSION-FREE SURVIVAL when compared to other studied groups [31]. You et al. compared two large independent datasets from GINECO and Gemelli Centre for predictive factors of successful interval cytoreductive surgery [32]. Multivariable logistic regression model for complete interval cytoreductive surgery (R0) showed the KELIM score (either as a continuous or dichotomized (favorable vs unfavorable) to be consistent significant prognostic factors between the two datasets. The radiological response score (CR-PR vs SD-PR) was only significant in the Gemelli database. A similar model was created for pathological response (CRS3), where a favorable KELIM score was significantly associated with better response in both datasets, and the radiological response was only significant in the GYNECO dataset. Even though the correlation between KELIM and CRS3 was significant in both of the datasets, there were large discrepancies in the odds ratios between the databases (OR = 21.44 [4.15–349.01 in GYNECO vs OR = 2.36 [1.51–3.70] in Gemelli database).

## Conclusions

This study evaluated the use of KELIM and RECIST criteria with regard to patients' prognosis. Our findings showed no significant associations between KELIM, RECIST and overall survival of patients. However, further studies on bigger homogenous population samples are required to confirm our findings.

## Data Availability

“The datasets generated during and/or analysed during the current study are available from the corresponding author on reasonable request.”
